# Genome-Wide Transcriptional Profiling Reveals MicroRNA-Correlated Genes and Biological Processes in Human Lymphoblastoid Cell Lines

**DOI:** 10.1371/journal.pone.0005878

**Published:** 2009-06-11

**Authors:** Liang Wang, Ann L. Oberg, Yan W. Asmann, Hugues Sicotte, Shannon K. McDonnell, Shaun M. Riska, Wanguo Liu, Clifford J. Steer, Subbaya Subramanian, Julie M. Cunningham, James R. Cerhan, Stephen N. Thibodeau

**Affiliations:** 1 Departments of Laboratory Medicine and Pathology, Mayo Clinic, Rochester, Minnesota, United States of America; 2 Health Sciences Research, Mayo Clinic, Rochester, Minnesota, United States of America; 3 Department of Genetics, Stanley S. Scott Cancer Center, Louisiana State University, New Orleans, Louisiana, United States of America; 4 Departments of Medicine and Genetics, Cell Biology, and Development, University of Minnesota, Minneapolis, Minnesota, United States of America; 5 Laboratory Medicine and Pathology, University of Minnesota, Minneapolis, Minnesota, United States of America; East Carolina University, United States of America

## Abstract

**Background:**

Expression level of many genes shows abundant natural variation in human populations. The variations in gene expression are believed to contribute to phenotypic differences. Emerging evidence has shown that microRNAs (miRNAs) are one of the key regulators of gene expression. However, past studies have focused on the miRNA target genes and used loss- or gain-of-function approach that may not reflect natural association between miRNA and mRNAs.

**Methodology/Principal Findings:**

To examine miRNA regulatory effect on global gene expression under endogenous condition, we performed pair-wise correlation coefficient analysis on expression levels of 366 miRNAs and 14,174 messenger RNAs (mRNAs) in 90 immortalized lymphoblastoid cell lines, and observed significant correlations between the two species of RNA transcripts. We identified a total of 7,207 significantly correlated miRNA-mRNA pairs (false discovery rate q<0.01). Of those, 4,085 pairs showed positive correlations while 3,122 pairs showed negative correlations. Gene ontology analyses on the miRNA-correlated genes revealed significant enrichments in several biological processes related to cell cycle, cell communication and signal transduction. Individually, each of three miRNAs (miR-331, -98 and -33b) demonstrated significant correlation with the genes in cell cycle-related biological processes, which is consistent with important role of miRNAs in cell cycle regulation.

**Conclusions/Significance:**

This study demonstrates feasibility of using naturally expressed transcript profiles to identify endogenous correlation between miRNA and miRNA. By applying this genome-wide approach, we have identified thousands of miRNA-correlated genes and revealed potential role of miRNAs in several important cellular functions. The study results along with accompanying data sets will provide a wealth of high-throughput data to further evaluate the miRNA-regulated genes and eventually in phenotypic variations of human populations.

## Introduction

Expression level of many mRNA genes shows abundant natural variation in human populations. The quantitative variations in mRNA expression are thought to contribute to phenotypic differences between individuals. Several molecular mechanisms have been identified that control gene expression. In addition to known transcription factors that bind to specific regulatory DNA sequences [1], [2] and extensively studied genetic polymorphisms that determine transcription level via *cis*- or *trans*-effects [3]–[8], newly discovered miRNAs have been proven to be a major player in posttranscriptional regulation of gene expression [1], [2], [9], [10]. The miRNAs were first identified to play a role in developmental timing of *Caenorhabditis elegans* in the early 1990s [11], [12]. Subsequent studies have shown that cellular factors necessary for miRNA biogenesis and many miRNAs are conserved in many organisms, suggesting the importance of miRNAs during developmental processes and evolutions [13]–[17].

miRNAs are a novel class of non-coding small RNAs which have been recognized as global regulators of gene expression that control the key cellular processes such as growth, development and apoptosis [9], [10]. A single miRNA can potentially regulate several hundreds of mRNAs forming a complex regulatory network that can act in a flexible manner for precise and rapid effects on protein translation and gene expression. Majority of the miRNAs are expressed in a cell- or tissue-specific manner and may contribute to the establishment and/or maintenance of cellular and/or tissue identity. It is estimated that several thousand human genes, up to about one-third of the mRNA transcriptome, are potential targets for regulation by miRNAs encoded in the genome [18]. The regulatory process occurs posttranscriptionally and involves miRNA interaction with a target site in the mRNA that has partial or complete complementarity to the miRNA. The regulatory effect of miRNAs on gene expression is a complex process involving both translational repression and accelerated mRNA turnover, each of which appears to occur by multiple mechanisms. Moreover, certain miRNAs are also capable of activating translation [19], [20]. Hence, miRNAs are related to diverse cellular processes and regarded as important components of the gene regulatory network.

Importance of an individual miRNA is reflected in the diseases that may arise upon the loss, mutation or dysfunction of specific miRNAs [21]–[23]. One study reported mutations in 5 of 42 sequenced miRNAs in 11 of 75 patients with chronic lymphocytic leukemia. Although the majority of these mutations were somatic, at least one was germline [23]. Another study showed that up-regulation of several miRNA genes was correlated with loss of their target gene transcript (KIT) in papillary thyroid carcinoma. In 5 of 10 such cases, this down expression was associated with germline single-nucleotide changes in the two recognition sequences in KIT for these miRNAs [22]. Recently, a series of papers presented conceptually related ideas linking the genetic variations and alterations of biogenesis and function of miRNAs to the increased risk of developing sixteen major human diseases. Significant role of miRNAs in the pathogenesis of many major human disorders has been proposed as part of disease phenocode concept [24]–[26]. These results suggest that germline changes in miRNAs and their target genes may have a profound effect not only on disease progression but also an individual's risk of developing disease.

Current studies, however, have focused primarily on miRNA role as posttranscriptional regulators of target mRNAs or at a much higher level on their cell biological processes and organismal roles [9], [10]. Loss- or gain-of-function studies often analyzed effects on mRNAs by expressing or suppressing specific miRNA in cells. As these experiments create non-physiological levels of miRNAs that may affect target mRNAs abnormally, accurate evaluation of the miRNA effects may require normal range of variations in miRNA expression under an endogenous condition. Additionally, because miRNAs can interact with their target genes directly and influence expressions of many other genes indirectly, the miRNAs may demonstrate correlations with their target genes as well as non-target genes. Therefore, merely measuring target gene expression may not be sufficient to gain understanding miRNA regulatory effects. A complimentary approach is to identify downstream genes that are tightly correlated with fluctuation of miRNA expression. Liu et al [27] has reported significant miRNA correlations with target genes as well as non-target genes by performing expression profiling analysis on 12 brain tumor biopsies. Subsequent experimental validations demonstrated a directional causal relationship from miRNAs to mRNAs. However, the small sample size and potential mutations in the samples restrained statistical power to detect weak correlations.

To fully examine the miRNA-mRNA correlations at whole genome scale, we measured both miRNA and mRNA transcriptional profiles in 90 human Epstein-Bar virus transformed lymphoblastoid cell lines. We performed pair-wise correlation coefficient analysis and identified strong correlations between the endogenous variations in the miRNA and mRNA expression. Gene Ontology (GO) analysis identified over-representation of these miRNA-correlated genes in several biological processes. These high-throughput expression data provides a valuable resource to examine global effects of miRNAs on gene expression and hence on complex traits.

## Results

### Endogenous correlations between miRNA and mRNA expression

**Figure 1:**
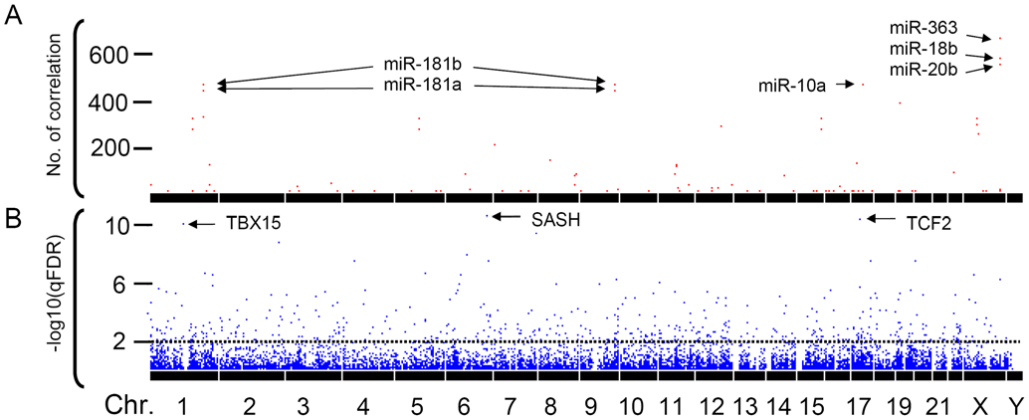
Frequency and significance level of miRNA-correlated genes. A. 90 of the 366 individual miRNAs are correlated with at least one mRNA. We align the 90 miRNAs on 20 of 24 chromosomes based on their genomic locations (X axis). For each miRNA, the number of the correlated mRNA probes is demonstrated on Y axis (red dot). For example, *miR-363* on Xq26.2 is correlated with 672 mRNA probes. Both *miR-181b* (correlated with 468 mRNA probes) and *miR-181a* (correlated with 440 mRNA probes) have two copies, each on different chromosomes (1 and 9). B. Among 11,417 known genes (14,174 mRNA probes), we successfully map 11,278 individual genes on the 24 chromosomes (X axis). We plot–log10 values of the correlation coefficient qFDRs between *miR-363* and the 11,278 genes along Y axis (blue dot). The horizontal dot line (black) indicates qFDR = 0.01. Above the line are the mRNA probes that were significantly correlated with the *miR-363*. For example, the *miR-363* is significantly associated with the gene *SASH* (chromosome 6q24.3) at qFDR = 3.70×10^−11^ (equal to 10.43 of −log scale in the figure).

We performed pair-wise correlation coefficient analysis to evaluate potential correlations between 366 miRNA and 14,174 mRNA expression levels. When false discovery rate q value (qFDR)<0.01 (approximately p value<0.00076), we detected significant correlation in 7,207 miRNA-mRNA pairs (**Table S1**), which were involved in 2,448 (17.27%) of the 14,174 mRNA probes and 90 (24.59%) of the 366 miRNAs. Of the 7,207 pairs, 4,085 and 3,122 pairs showed positive and negative correlations, respectively (**Table 1**, also see **Table S2** for all 366*14,174 correlation coefficient r values). The most frequently involved miRNA was *miR-363*, which was correlated with 672 mRNAs. Cumulative frequency of these correlated genes for each of 366 miRNAs is shown in **Figure 1A**. Significance level of each mRNA probe for its correlation with *miR-363* is demonstrated in **Figure 1B**.

**Table 1:** Correlation between miRNAs and mRNAs in lymphoblastoid cell linesΔ.

		Bonferroni-corrected p<0.05			qFDR<0.01		
		Positive correlation	Negative correlation	Any Correlation	Positive correlation	Negative correlation	Any Correlation
miRNA-mRNA pairs		204	15	219	4085	3122	7207
mRNAs involvedΔΔ		103	14	116	1453	1417	2448
miRNAs involvedΔΔ		26	13	30	72	64	90
Top 20 miRNA-correlated mRNAs							
	miR-363	33	0	33	383	289	672
	miR-18b	33	1	34	370	214	584
	miR-20b	34	0	34	349	207	556
	miR-181b	10	2	12	304	164	468
	miR-10a	13	1	14	259	208	467
	miR-181a	18	1	19	267	173	440
	miR-181c	8	0	8	243	144	387
	miR-213	9	0	9	199	125	324
	miR-221	11	0	11	196	125	321
	miR-9*	1	1	2	136	184	320
	miR-222	9	1	10	173	122	295
	miR-331	0	1	1	84	200	284
	miR-9	2	1	3	111	160	271
	miR-98	0	2	2	93	159	252
	miR-339	2	0	2	111	92	203
	miR-486	2	0	2	80	56	136
	miR-33b	0	0	0	85	41	126
	miR-194	4	0	4	83	38	121
	miR-192	2	0	2	76	38	114
	miR-130b	0	0	0	47	38	85

Δ The number in the table are mRNA probe counts.

ΔΔ Because one miRNA (mRNA) may be correlated with one mRNA (miRNA) positively and another mRNA (miRNA) negatively, number of any correlation is smaller than sum of positive and negative correlations.

When examining each of 7,207 significant miRNA-mRNA pairs individually, we found that positive correlations dominated highly correlated pairs. The positive correlations accounted for top 110 pairs (ranked based on qFDR values). The correlation between *miR-10a* and *HOXB4* was the most significant with a positive correlation qFDR = 2.21×10^−19^. The most significant negative correlation was between *miR-98* and *SERBP1* (qFDR = 3.97×10^−7^), which was ranked 111^th^ of the most significant pairs. **Table 2** lists top 20 most significant positive and top 20 negative correlation pairs.

**Table 2:** Top 20 miRNA-mRNA pairs (based on correlation qFDR values).

	miRNAs	mRNA genes	r	p	Bonferroni-corrected p	Correlation qFDR	Co-localization
**Top 20 positively correlated pairs**							
	miR-10a	HOXB4	0.823	2.63E-23	1.36E-16	2.21E-19	yes
	miR-151	PTK2	0.795	8.10E-21	4.21E-14	9.30E-17	yes
	miR-28	LPP	0.791	1.92E-20	9.96E-14	1.99E-16	yes
	miR-18b	SASH1	0.757	6.16E-18	3.20E-11	5.36E-14	no
	miR-20b	SASH1	0.739	9.51E-17	4.94E-10	8.62E-13	no
	miR-10a	HOXB2	0.720	1.23E-15	6.37E-09	5.18E-12	yes
	miR-18b	TCF2	0.718	1.62E-15	8.42E-09	7.06E-12	no
	miR-20b	TBX15	0.718	1.60E-15	8.31E-09	7.24E-12	no
	miR-222	ACVR1B	0.722	9.62E-16	4.98E-09	9.77E-12	no
	miR-18b	TBX15	0.707	6.94E-15	3.60E-08	2.01E-11	no
	miR-363	SASH1	0.715	2.61E-15	1.35E-08	2.30E-11	no
	miR-424	MGC16121	0.716	2.10E-15	1.09E-08	2.62E-11	yes
	miR-20b	TCF2	0.702	1.30E-14	6.73E-08	3.93E-11	no
	miR-363	TCF2	0.705	8.97E-15	4.65E-08	3.96E-11	no
	miR-20b	BC062771	0.698	2.11E-14	1.09E-07	4.78E-11	no
	miR-363	TBX15	0.695	2.97E-14	1.54E-07	8.73E-11	no
	miR-20b	HLXB9	0.683	1.27E-13	6.59E-07	2.31E-10	no
	miR-221	ACVR1B	0.693	3.64E-14	1.89E-07	3.39E-10	no
	miR-363	HLXB9	0.680	1.63E-13	8.45E-07	3.60E-10	no
	miR-222	RBMS1	0.686	8.11E-14	4.21E-07	4.12E-10	no
**Top 20 negatively correlated pairs**							
	miR-98	SERBP1	−0.625	4.58E-11	0.0002	3.97E-07	no
	miR-18b	C13orf18	−0.58	2.11E-09	0.0109	7.04E-07	no
	miR-181b	CKLF	−0.586	1.27E-09	0.0066	1.95E-06	no
	miR-363	SMARCA2	−0.557	1.16E-08	0.0600	2.63E-06	no
	miR-10a	CUGBP2	−0.570	4.58E-09	0.0238	2.76E-06	no
	miR-363	CHST2	−0.554	1.52E-08	0.0787	3.27E-06	no
	miR-222	DDR2	−0.577	2.58E-09	0.0134	3.27E-06	no
	miR-18b	CUGBP2	−0.550	1.99E-08	0.1032	4.22E-06	no
	miR-363	TGFBR3	−0.548	2.21E-08	0.1146	4.24E-06	no
	miR-18b	SMARCA2	−0.549	2.06E-08	0.1069	4.27E-06	no
	miR-363	BAG3	−0.547	2.35E-08	0.1219	4.42E-06	no
	miR-181a	BPNT1	−0.562	8.39E-09	0.0436	4.50E-06	no
	miR-181b	ST7	−0.571	4.34E-09	0.0225	5.12E-06	no
	miR-363	ARL8B	−0.544	2.90E-08	0.1504	5.22E-06	no
	miR-18b	AP1S3	−0.545	2.71E-08	0.1402	5.35E-06	no
	miR-331	E2F2	−0.596	5.67E-10	0.0029	5.38E-06	no
	miR-151	VIM	−0.590	9.67E-10	0.0050	5.55E-06	no
	miR-18b	C22orf16	−0.544	2.99E-08	0.1548	5.65E-06	no
	miR-363	KIF3B	−0.542	3.45E-08	0.1790	5.67E-06	no
	miR-363	SAV1	−0.540	3.82E-08	0.1984	6.14E-06	no

To confirm and validate the Illumina BeadArray data, we tested 6 miRNAs (*miR-10a, -20b, -181b, -181c, -34b, -372*) and 7 mRNA genes (*GRK5, KIF3B, ADD3, HOXB4, SERBP1, ST7* and *ZNF532*) using TaqMan-based quantitative RT-PCR in each of the 90 lymphoblastoid cell lines. The 6 miRNAs were chosen because they demonstrated various degrees of correlations with mRNAs and were in the same multiplex RT pool (human pool 1) of TaqMan miRNA assays. The 7 mRNA genes were selected based on various levels of correlations with the selected miRNAs. Four of the six miRNAs (*miR-10a, -20b, -181b, -181c*) and all 7 mRNA genes had a Ct (cycle threshold) value≤35 and so were used in our final analysis. The results showed high level of concordance between the BeadArray and quantitative RT-PCR in 26 of the 28 comparisons (**Table 3**). Two miRNA-mRNA pairs (*miR-20b* and *ST7*, and *miR-181c* and *ST7*) gave poor reproducibility, and this may have resulted from the use of the two separate assays in targeting different isoforms (*a* and *b*) of the gene *ST7*.

**Table 3:** Confirmation of beadarray data with TaqMan-based quantitative RT-PCRΔ.

Genes	*miR-10a*		*miR-20b*		*miR-181b*		*miR-181c*	
	Beadarray	qRT-PCR	Beadarray	qRT-PCR	Beadarray	qRT-PCR	Beadarray	qRT-PCR
***GRK5***	−0.012	−0.060	0.024	0.027	−0.172	−0.027	−0.147	0.178
***KIF3B***	−0.483	−0.469	−0.509	−0.540	−0.529	−0.391	−0.532	−0.229
***ADD3***	0.113	0.158	0.201	0.404	0.436	0.514	0.399	0.404
***HOXB4***	0.823	0.773	0.503	0.524	0.348	0.547	0.345	0.375
***SERBP1***	0.062	0.067	0.192	0.131	0.125	0.051	0.131	0.204
***ST7***	−0.364	−0.268	−0.440	−0.096	−0.571	−0.337	−0.529	−0.083
***ZNF532***	0.307	0.290	0.218	0.261	0.276	0.259	0.228	0.215

Δ Spearman rank correlation coefficients (r value) between these miRNA and mRNA gene expressions are used for the comparison. Among the 28 comparisons, 26 show high concordance. One discordant pair is the gene *ST7* and *miR-20b* with an r value of −0.440 in Beadarray and −0.096 in qRT-PCR. Another pair is the gene *ST7* and *miR-181c* with an r value of −0.529 in Beadarray and −0.083 in qRT-PCR. The two different assays that target different isoforms (*a* and *b*) of the *ST7* is a plausible explanation.

### miRNA-correlated genes and biological processes

To functionally classify miRNA-correlated genes, we used 11,417 known genes (14,174 mRNA probes) as a reference set and applied GOMiner (http://discover.nci.nih.gov/gominer/) for enrichment analysis of selected gene sets. We first evaluated GO classification for each miRNA-correlated gene set. To correct for multiple comparisons, we performed 1000 randomization analyses. For each of these gene sets, we observed various degrees of GO term enrichments. For example, eight of the top 20 miRNAs in **Table 1** had at least one GO term that was significantly over-represented (randomization-corrected FDR<0.01). The most striking findings were from gene sets that were correlated with *miR-331, miR-98* and *miR-33b*. For *miR-331*-correlated genes (269 genes from 284 probes), we detected significant over-representation for the GO terms of DNA replication (p = 2.65×10^−23^, 8.67 fold enrichment), DNA metabolic process (p = 2.35×10^−22^, 4.15 fold enrichment) and cell cycle (p = 1.94×10^−19^, 3.66 fold enrichment). Further analysis demonstrated that these enrichments were exclusively derived from negatively correlated genes. While we did not see any significant GO term in positively correlated genes, we observed significant enrichments in 58 GO terms for 191 negatively correlated genes (200 probes). Again, the most marked GO term included DNA replication (p = 9.21×10^−28^, 11.59 fold enrichment), cell cycle (p = 7.99×10^−27^, 4.89 fold enrichment) and DNA metabolic process (p = 1.09×10^−26^, 5.34 fold enrichment) (**Table 4**).

**Table 4:** Gene Ontology analysis of miRNA-correlated genesΔ.

	GO ID	GO terms	Total Genes	miRNA-correlated Genes	Enrichment Fold	Enrichment p value	Enrichment qFDRΔΔ
*miR-331* **negatively** associated genes (191 genes from 200 probes)							
	GO:0006260	DNA replication	142	34	11.59	9.21E-28	0
	GO:0007049	Cell cycle	574	58	4.89	7.99E-27	0
	GO:0006259	DNA metabolic process	489	54	5.34	1.09E-26	0
	GO:0022402	Cell cycle process	489	49	4.85	4.31E-22	0
	GO:0022403	Cell cycle phase	214	31	7.01	2.02E-18	0
*miR-98* **negatively** associated genes (153 genes from 159 probes)							
	GO:0007049	Cell cycle	574	38	4.12	6.01E-15	0
	GO:0000278	Mitotic cell cycle	201	22	6.80	4.97E-13	0
	GO:0022403	Cell cycle phase	214	22	6.39	1.81E-12	0
	GO:0006259	DNA metabolic process	489	32	4.07	2.22E-12	0
	GO:0022402	Cell cycle process	489	32	4.07	2.22E-12	0
*miR-33b* **positively** associated genes (84 genes from 85 probes)							
	GO:0000279	M phase	176	12	8.04	2.16E-08	0
	GO:0007049	Cell cycle	574	20	4.11	2.88E-08	0
	GO:0007067	Mitosis	146	11	8.88	3.17E-08	0
	GO:0000087	M phase of mitotic cell cycle	148	11	8.76	3.65E-08	0
	GO:0051301	Cell division	163	11	7.95	9.91E-08	0
All miRNA **positively** associated genes (1,206 genes from 1,453 probes) Δ Δ Δ							
	GO:0007154	Cell communication	1553	251	1.36	6.85E-09	0
	GO:0007165	Signal transduction	1430	229	1.34	1.05E-07	0
	GO:0002376	Immune system process	401	81	1.70	6.30E-07	0
	GO:0032501	Multicellular organismal process	1143	182	1.34	5.37E-06	0.0005
	GO:0006955	Immune response	307	63	1.72	6.99E-06	0.0004

Δ only top five GO terms are listed here. For other enriched GO terms, please see **Table S3**.

ΔΔ randomization-corrected FDRs are reported here. Refer to Materials and Methods for detail.

ΔΔΔ the gene list has excluded the genes that are correlated with *miR-331, miR-98* and *miR-33b*.

For the gene sets that were correlated with *miR-98* (239 genes from 348 probes) and *miR-33b* (124 genes from 126 probes), we observed similar GO term enrichments as the *miR-331* correlated genes. This was particularly true for the GO terms related to cell cycle. When considering both types of the correlated genes separately, however, we found the cell cycle-related GO term enrichments only in the *miR-98* negatively correlated (153 genes from 159 probes) and in the *miR-33b* positively correlated gene sets (84 genes from 85 probes) (**Table 4**).

We then evaluated overall influence of miRNA expression on the GO categories. For a total of 2,248 miRNA-correlated genes (from 2,448 mRNA probes), we detected significant enrichments on several GO terms, in particular those pertinent to cell cycle with 72% (8 of 11 significantly enriched terms) relevant. Other significant GO terms included those related to cell communication, signal transduction and response to stimulus. To see if these cell cycle-related enrichments were driven by the *miR-331*, *miR-98* and *miR-33b*, we excluded genes that were correlated with these 3 miRNAs and re-evaluated the GO term distribution. We found that cell cycle-related terms were no longer over-represented, but the two terms (cell communication and signal transduction) still remained enriched. Interestingly, further analysis identified that only positively correlated gene set contributed to the enrichments with p = 6.85×10^−9^ for cell communication and p = 1.05×10^−7^ for signal transduction (**Table 4**). Other enriched terms included immune system process (p = 6.30×10^−7^) and multicellular organismal process (p = 5.37×10^−6^). **Table S3** provides these significant GO terms with randomization-corrected FDR<0.01 in detail.

### Direct vs. indirect miRNA-mRNA correlations

To test if miRNA-correlated mRNA genes are direct miRNA targets, we downloaded the predicted miRNA targets from TargetScan5.1 (http://www.targetscan.org) and compared them with the miRNA-correlated genes. Because miRNA annotation file was based miRBase v9.1, some of miRNA names did not match the new version of TargetScan such as missing the -3p or -5p. Therefore, we limited our analysis to these miRNAs with name or sequence match in the TargetScan 5.1. We also limited the analysis to these genes with a reference sequence accession number. Before statistical analysis, we further filtered out the miRNAs that had less than 10 correlated mRNAs. Finally, 31 miRNAs were left for target prediction. We then compared each list of miRNA-correlated gene set to the list of predicted miRNA target genes. For the 31 miRNAs, we found 8 miRNAs whose targets were significantly enriched among their correlated gene sets (p<0.05 when included both conserved and non-conserved targets). The target gene set of miR-181a remained significant after multiple testing correction (FDR = 0.048).

### Physical proximity and expression correlations

**Figure 2:**
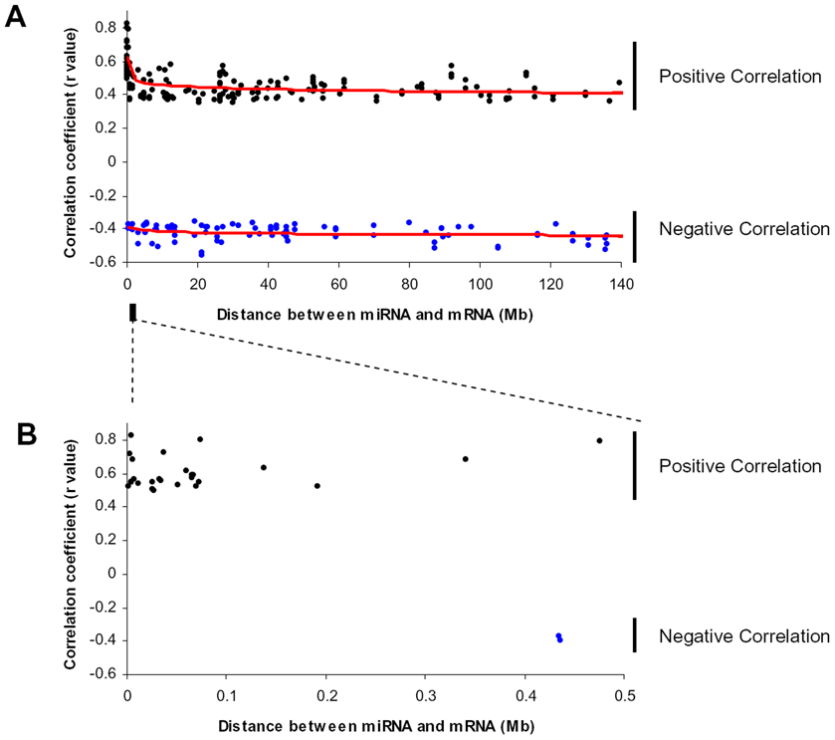
Physical proximity and expression correlations. We extract all miRNA-mRNA pairs that map on same chromosomes and have correlation qFDR<0.01. We plot the physical distances between miRNAs and mRNAs on x-axis and correlation coefficient r values on y-axis. The distance is based on the starting position of each miRNA and mRNA. We draw a logarithmic trend line (red) for positive and negative correlations separately. A. the distances from near 0 to 140 Mb are shown. For positive correlations (black dots), the trend line shows significant r value drop at far left side (short distance). However, for negative correlations (blue dots), the trend line tends to be constant. B. the distance from 0 to 0.5 Mb was shown. The positive correlations (26 pairs) are clearly clustered when the distance is <0.4 Mb (particularly <0.1 Mb), where no negative correlation is found.

To estimate the effect of miRNA-mRNA proximity on their expression correlation, we extracted all miRNA-mRNA pairs that mapped on the same chromosomes and had correlation qFDR<0.01. We plotted r value of each correlation against distance between the miRNA and mRNA (**Figure 2**). For positive correlations, we observed clear trend of r value decrease when the distance increased. Specifically, the highest positive correlations were observed when miRNA and its corresponding mRNA was physically close each other on a chromosome. The highly positive correlations gradually decreased to baseline (at ∼≥2 Mb). For negative correlations, however, we observed constant r values from 0.4 Mb to over 140 Mb. We did not observe any correlation when the distance was <0.4 Mb, where 26 positive correlations existed.

## Discussion

miRNAs play an important role in regulation of gene expression. In this study, we examined genome-wide expression profiling in normal lymphoblastoid cell lines under routine culture condition and identified strong correlations between miRNA and mRNA expressions. Although complex gene-gene interactions may greatly diminish the power to identify significant miRNA effects, we were able to detect a variety of biological processes that may indicate function of those miRNAs. These results demonstrate that genome-wide transcriptional profiling analysis was able to detect endogenous correlations between miRNA and mRNA and that miRNA regulatory effect was discernable under natural culture condition.

miRNAs have been shown to target transcripts that encode proteins involved in cell cycle progression and cellular proliferation. Some of them display defective expression patterns in human tumors with the consequent alteration of target oncogene or tumor suppressor genes. These miRNAs modulate the major proliferation pathways through direct interaction with critical regulators such as *MYC, RAS, PI3K/PTEN* or *ABL*, as well as members of the retinoblastoma pathway, *Cyclin-CDK* complexes or cell cycle inhibitors of the *INK4* or *Cip/Kip* families[28], [29]. It is postulated that by regulating an entire cellular program through the cooperative repression of target genes, miRNAs may serve as buffers to limit the accumulation of many gene products that impact cell cycle progression under a variety of contexts[28].

Interestingly, the *miR-98* in this study is one of miRNAs that show significant association with cell cycle-related genes. The *miR-98* is a member of *let-7* family which plays a critical role in cell cycle control with respect to differentiation and tumorigenesis. The *let-7* family is a master regulator of cell proliferation pathways by regulating the expression of the *RAS* as well as *MYC* oncogenes[30]–[32]. Over-expression of *let-7* miRs alters cell cycle progression and reduces cell division in lung cancer cells[30] and causes cell cycle arrest by directly regulating the gene *Cdc34* in human fibroblasts[33]. Clearly, the *let-7* is a negative regulator of cell cycle process, which is consistent with our observation that *miR-98* is negatively correlated with cell cycle genes in this study. Additionally, we also noticed a correlation of other *let-7* members (especially *let-7i* and *let-7g*) with cell cycle-related genes (**Table S1**) in the lymphoblastoid cell lines, suggesting that *let-7* family members do regulate cell cycle not only in fibroblasts and cancer cells but also in lymphoblastoid cell lines.

Because direct regulation of gene expression by miRNAs, we expect to see enrichment of miRNA-correlated genes among predicted targets. Indeed, we observed significant concordance between miRNA-correlated genes and miRNA predicted target genes in 8 of 31 miRNA sets. In particular, the miR-181a gene set showed significance of concordance even after multiple testing correction. For some reasons, we did not see any evidence of concordance in most miRNA sets. Generally, miRNA is believed to bind 3′UTR of a target gene and regulates gene expression at protein level. Therefore, miRNA target itself may not demonstrate noticeable change at mRNA level although some exceptions have been reported[34]. It is especially true when these correlations are examined under endogenous condition and there is limited range of variations in miRNA expression (contrary to extremely high level of expression by transfection study). Because miRNA inhibitory effect is at posttranscriptional level, the most significant changes could be downstream genes of the miRNA target at transcript level. Furthermore, because background noise and weak correlation between miRNAs and their targets, each tested miRNA had relatively small number of correlated genes when applying FDR<0.01. This also limited statistical power to detect significance.

Although the results from this study need further validation, the importance of the present study is clear. First, the study adopts a whole genome approach and identifies thousands of highly correlated miRNA-mRNA pairs. Second, the study measures expression levels under endogenous condition and without extremely high or low levels of miRNAs caused by transfection approach. Third, the study correlates miRNAs with their targets as well as downstream genes. The downstream genes are closer to the final consequences of miRNA-mRNA interactions. Fourth, the study identifies several biological processes that are associated with variations in miRNA expression. Lastly, the study results are supported by other publications using different materials and methods, demonstrating feasibility of this approach in studying miRNA function. We believe that these results along with supplementary data sets will provide a valuable resource to further investigate the miRNAs and their functions.

## Materials and Methods

### Ethics Statement

All subjects provided written informed consent; and the study was approved by the Mayo Clinic IRB.

### Cell lines and Cell culture

We collected peripheral bloods from 90 Caucasian men with median age of 68 years old (44–74) and transformed the peripheral blood lymphocytes with Epstein-Bar virus to establish immortal cell lines. We then grew all transformed cell lines in RPMI 1640 media supplemented with 15% fetal bovine serum, and 1% penicillin/streptomycin at 37°C in humidified incubators in an atmosphere of 5% CO_2_. Experimental series were set up by seeding 5-ml cultures in T25 flasks. Each culture was fed with 5 ml of fresh media twice a week until the cell number reached ∼10^6^ in a T75 flask. The cells were harvested and suspended in 500 µl of RNAlater and stored at −80°C for further processing.

### RNA extraction

We extracted total RNA from each cell culture using miRNeasy Mini Kit (QIAGEN) under the manufacturer's guidelines. This protocol could effectively recover both mRNA and miRNA. The integrity of these total RNAs was assessed using an Agilent 2100 Bioanalyzer.

### mRNA and miRNA microarrays

We used the Illumina human-6 V2 BeadChip for mRNAs profiling and Illumina microRNA expression profiling panel (based on miRbase release 9.0) for miRNA analysis according to the manufacturer's recommendation (Illumina, Inc., San Diego, CA). 200 ng of RNA from each cell culture was first labeled and then hybridized to each array using standard Illumina protocols. BeadChips (mRNA) or sample array matrices (miRNA) were scanned on an Illumina BeadArray reader. For mRNA, we repeated 30 samples in triplicate, 30 samples in duplicate and 30 singleton samples for a total of 180 expression profiles. For miRNA, we repeated 84 samples in duplicate and 6 samples in quadruplicate for a total of 192 expression profiles. We also arranged each of these replicates in separate arrays to reduce potential batch effect. Before data processing, we used various bioinformatics tools to examine the quality and reproducibility of each expression profiles. Based on principal component analysis, we removed 26 individual miRNA profiles due to substantial shifts away from a main cluster. However, replicates from each of the 26 individuals were still included in the analysis because they were in the main cluster. The expression profiles have been deposited in NCBI's Gene Expression Omnibus (GEO) with accession number GSE14794.

### Data processing

We processed 180 mRNA and 166 miRNA profiles which included all 90 subjects. For both mRNA and miRNA data, we first transformed raw data generated from BeadStudio (Illumina, San Diego, CA) using a variance stabilization transformation algorithm [35] and then normalized them using quantile normalization implemented in Bioconductor (www.bioconductor.org). After normalization, the samples with replicates were averaged. As a large fraction of mRNAs and miRNAs were either not expressed or non-detectable, we filtered out probes with median detection p value≥0.01 (the p values were automatically reported in BeadStudio). This procedure reduced the number of mRNA probes from 48702 to 14174 and of miRNA probes from 736 to 366 for final data analysis. Among the 366 miRNAs, 273 are in miRBase database (http://microrna.sanger.ac.uk, v9.1), and 93 are potential miRNAs identified in a RAKE analysis[36], [37].

### Data analysis

For each of the 366 miRNAs, we correlated them with 14,174 mRNA probes in the 90 individuals using Spearman's rank correlation analyses. We assessed statistical significance using q value of false discovery rate (qFDR) based on Storey et al [38] and Bonferroni correction. In this study, we defined the miRNA-mRNA correlation coefficient qFDR<0.01 to be statistically significant. We also reported our findings using Bonferroni-corrected p<0.05 (an equivalent to un-corrected p<9.64×10^−9^, 0.05 divided by 366×14,174). These analyses were done using Partek Genomics Suite (Partek Inc, St. Louis, Missouri). To explore whether miRNA affects expression of genes that share a common biological relationship, we searched for over-representation in GO categories from miRNA-correlated genes. We labeled genes with positive correlation (+1) and genes with negative correlation (−1). One gene list for each miRNA was submitted to the High-Throughput GoMiner at http://discover.nci.nih.gov/gominer/. The reference gene list was 11,417 known gene names from the 14,174 mRNA probes. The GO annotation was obtained by matching to the gene names in the UniProt database. We specified 1,000 randomizations for calculating the GO enrichment FDR q-value. The enrichment p-value for each GO category was calculated using Fisher exact test and the q-value was calculated using the distribution of the p-values obtained by randomly re-sampling from the reference genes[39], [40].

### Real-Time PCR analyses of mRNAs and miRNAs

For mRNAs, 1.2 ug of total RNAs from each of the 90 subjects was converted to cDNA by High Capacity RNA-cDNA Master Mix (Applied Biosystems, Foster City, CA) in a 40 ul reaction according to the manufacturer's protocol. 1 ul of the cDNA was used for real time quantitative PCR in a total volume of 15 ul containing 1× TaqMan Gene Expression Master Mix and gene specific assay for selected genes which included *GRK5* (Hs00992173), *KIF3B* (Hs01122781_m1), *ADD3* (Hs00249895_m1), *HOXB4* (Hs00256884_m1), *SERPB1* (Hs00854675_gH), *ST7* (Hs00251157_m1), *ZNF532* (Hs00539543_m1) and *GADPH* (Applied Biosystems). For miRNAs, 0.4 ug of total RNAs was converted to cDNA using TaqMan MicroRNA Reverse Transcription Kit and multiplex RT primer pool 1. 1.8 ul of 1∶10 diluted cDNAs was used for real time quantitative PCR in a total volume of 15 ul containing 1× TaqMan Universal PCR Master Mix and specific assay for selected miRNAs which included *miR-10a, miR-20b, miR-181b, miR181c, miR-34b, miR-372* and *RUN48* (Applied Biosystems). All PCR assays were run in triplicate, and expression values were averaged. In order to increase the reliability, we excluded the assays with Ct value≥35, which included the miRNA assays for *miR-34b and miR-372*. To calculate the relative expression for each transcript, all real-time PCR data were normalized to GAPDH (mRNA) or RUN48 (miRNA) by ΔCt method. We also converted the ΔCt into a value of 20-ΔCt such that the latter value was positively proportional to the log of copy number and was comparable with log transformed data from microarrays.

## Supporting Information

Table S1 Excel spreadsheet containing all miRNA-mRNA pairs with significant correlation (qFDR<0.01).
(3.28 MB XLS)

Table S2 Pairwise correlation coefficient r values between 366 miRNAs and 14,174 mRNAs.
(19.72 MB ZIP)

Table S3 Excel spreadsheet listing gene ontology analysis of miRNA-correlated genes.
(0.06 MB XLS)
